# Vaginismus

**Published:** 1876-02

**Authors:** 


Dr. Lutland, in *These de Paris,* concludes that vaginismus is always symptomatic. It is a common affection, which manifests itself chiefly after the first sexual congress. Vaginismus is often allied to dysmenorrhœa, and to the troubles of general innervation. It is always curable, the cure being easier if the malady is recent. By the obstacle which it offers to the accomplishment of sexual functions, vaginismus is a frequent cause of sterility. The treatment of vaginismus is simple—cauterization and dilatation are the principal means to overcome it.

